# Discordance between PAM50 intrinsic subtyping and immunohistochemistry in South African women with breast cancer

**DOI:** 10.1007/s10549-023-06886-3

**Published:** 2023-03-03

**Authors:** Thérèse Dix-Peek, Boitumelo P. Phakathi, Eunice J. van den Berg, Caroline Dickens, Tanya N. Augustine, Herbert Cubasch, Alfred I. Neugut, Judith S. Jacobson, Maureen Joffe, Paul Ruff, Raquel A. B. Duarte

**Affiliations:** 1 Department of Medicine, Faculty of Health Sciences, University of the Witwatersrand, 7 York Road, Johannesburg, 2193 South Africa; 2 Department of Surgery, School of Clinical Medicine, Faculty of Health Sciences, University of Kwa-Zulu Natal, Durban, 4001 South Africa; 3 Department of Surgery, Faculty of Health Sciences, University of the Witwatersrand, 7 York Road, Johannesburg, 2193 South Africa; 4 Department of Histopathology, National Health Laboratory Service, Chris Hani Baragwanath Hospital, 26 Chris Hani Road, Diepkloof, Johannesburg, 1864 South Africa; 5 Department of Anatomical Pathology, University of the Witwatersrand, 7 York Road, Johannesburg, 2193 South Africa; 6 School of Anatomical Sciences, Faculty of Health Sciences, University of the Witwatersrand, 7 York Road, Parktown, Johannesburg, 2193 South Africa; 7 Batho Pele Breast Unit, Chris Hani Baragwanath Academic Hospital, 26 Chris Hani Road, Diepkloof, Soweto, 1860 South Africa; 8 SA MRC Common Epithelial Cancer Research Centre, Faculty of Health Sciences, University of the Witwatersrand, Johannesburg, 2193 South Africa; 9 Herbert Irving Comprehensive Cancer Centre, Vagelos College of Physicians and Surgeons, Columbia University, New York, NY 10032 USA; 10 Department of Epidemiology, Mailman School of Public Health, Columbia University, New York, NY 10032 USA; 11 Strengthening Oncology Services Research Unit, Faculty of Health Sciences, University of the Witwatersrand, Johannesburg, 2193 South Africa

**Keywords:** Breast cancer, Sub-Saharan Africa, Subtypes, Immunohistochemistry, PAM50

## Abstract

**Purpose:**

Breast cancer is a heterogeneous disease with different gene expression profiles, treatment options and outcomes. In South Africa, tumors are classified using immunohistochemistry. In high-income countries multiparameter genomic assays are being utilized with implications for tumor classification and treatment.

**Methods:**

In a cohort of 378 breast cancer patients from the SABCHO study, we investigated the concordance between tumor samples classified by IHC and the PAM50 gene assay.

**Results:**

IHC classified patients as ER-positive (77.5%), PR-positive (70.6%), and HER2-positive (32.3%). These results, together with Ki67, were used as surrogates for intrinsic subtyping, and showed 6.9% IHC-A-clinical, 72.7% IHC-B-clinical, 5.3% IHC-HER2-clinical and 15.1% triple negative cancer (TNC). Typing using the PAM50 gave 19.3% luminal-A, 32.5% luminal-B, 23.5% HER2-enriched and 24.6% basal-like. The basal-like and TNC had the highest concordance, while the luminal-A and IHC-A group had the lowest concordance. By altering the cutoff for Ki67, and realigning the HER2/ER/PR-positive patients to IHC-HER2, we improved concordance with the intrinsic subtypes.

**Conclusion:**

We suggest that the Ki67 be changed to a cutoff of 20–25% in our population to better reflect the luminal subtype classifications. This change would inform treatment options for breast cancer patients in settings where genomic assays are unaffordable.

**Supplementary Information:**

The online version contains supplementary material available at 10.1007/s10549-023-06886-3.

## Introduction

Breast cancer is the most commonly diagnosed cancer among South African women accounting for 27.1% of all cancers diagnosed in these women [1]. Breast cancer diagnoses on the African continent have been steadily increasing over the past decades, attributed to longer lifespans and changes in lifestyle associated with westernization. In Africa, mortality rates are higher than in Europe and the United States, largely due to late stage at diagnosis and fewer treatment options [2, 3]. Breast cancer is a heterogeneous disease, differing in gene expression patterns, growth rates, responses to treatment and clinical outcomes.

Breast tumors can be subtyped by immunohistochemistry (IHC) which investigates the expression of four biomarkers: estrogen receptor (ER), progesterone receptor (PR), human epidermal growth factor receptor 2 (HER2/neu) and a marker of proliferation, Ki67. These markers distinguish ER-positive A-like, ER-positive B-like; HER2-enriched and triple negative (TNC) tumors (Table 1) [4–6]. Analysis of expression of the hormone receptors (ER and PR) is a semi-quantitative method based on the Allred Score [7–9].

**Table 1:** Immunohistochemical surrogates for expected PAM50 Intrinsic subtypes, showing IHC used for clinical decisions and Ki67 cutoff values for concordance analysis

PAM50	IHC clinical*		IHC analysis	
Intrinsic subtype	Designation	IHC expression	Designation	IHC expression
Luminal-A	Clin-A	HR + / HER2− / Ki67 ≤ 14%	A-like	HR + / HER2− / Ki67 ≤ 10%
			A- or B- like	HR + / HER2− / 10% < Ki67 ≤ 30%
Luminal-B	Clin-B	HR + / HER2− / Ki67 > 14%		
			B-like	HR + / HER2− / Ki67 > 30%)
		HR + / HER2 + / Ki67 any		
HER2-enriched			B/HER2-like	HR + / HER2 + / Ki67 any
	Clin-HER2	HR− / HER2 + / Ki67 any	HER2-like	HR− / HER2 + / Ki67 any
Basal-like	Clin-TNC	HR− / HER2− / Ki67 any	TNC	HR− / HER2− / Ki67 any

*Current multidisciplinary tumour board guidance algorithms for treatment decision making

*HR* hormone receptor, indicates either estrogen receptor and/or progesterone receptor; *HER2* human epidermal growth factor receptor 2, *TNC* triple negative cancer, + , positive; −, negative

The proliferation marker Ki67 is used to distinguish between the luminal subtypes [10, 11] and was adopted as a marker by the St Gallen International Consensus on Breast Cancer [5]. Ki67 was introduced as a diagnostic marker in South Africa in 2013 and is indicative of proliferation if Ki67 expression is ≥ 14% [5, 10]. However, the optimal Ki67 cut off value to distinguish luminal A-like tumors from luminal B-like tumors remains controversial due to uncertainty about how to classify tumors with intermediate (10–30%) Ki67 levels [12]. The 2015 St Gallen’s suggested a cutoff of 20–29% be used to distinguish A-like and B-like subtypes, along with clinical validation [13]. In addition, IHC for Ki67 analysis lacks reproducibility across laboratories [14]. Immunohistochemical results can be affected by the duration of fixation, type of fixative used, speed of assay and completeness of dehydration [15]. Moreover, the assessment is subject to interpretation by the histopathologist. In South Africa, the Department of Health 2018 recommendations are to use a Ki67 cutoff of 14% [16], although there is ongoing debate to the best cutoff of Ki67 to distinguish between luminal subtypes [17]. Ki67 cutoffs of both 14% and 20% are currently used at different centers.

The last decade has seen the development of many commercialized multigene tests to guide treatment and provide prognostic information for patients with breast cancer. The PAM50/ Prosigna assay has a 50-gene signature that groups tumors into intrinsic molecular subtypes luminal-A, luminal-B, HER2-enriched and basal-like [18]. The PAM50 assay is less subjective than the IHC-based techniques, but is much more expensive and labor intensive than IHC. In South African public hospitals, IHC continues to be used for clinical subtyping because of its lower cost.

A recent study from South Africa found that 64.9% of patients were diagnosed by IHC4 as B-like, 15.3% as TNC, 13.8% as A-like, and 6.0% as HER2-enriched [19]. An earlier country-wide study, found that black South African women had higher levels of ER-negative and PR-negative tumors than women of European, South Asian or admixture heritage, but did not have significantly different HER2 levels [15]. More recently, a study showed that white South African women had similar IHC profiles to European women and white American women, with more aggressive subtypes predominant in young women and less aggressive subtypes in older women, whereas black South African women did not have substantial profile changes according to age [20].

This study examines the concordance between PAM50 molecular subtyping assigned and the IHC results currently used for the management of breast cancer diagnosed within the South African Public Health System, focusing on varying Ki67 cutoffs. The data generated should help to inform cutoff values for IHC and may lead to better management of breast cancer in South Africa and other settings where genomic subtyping is unaffordable.

## Methods

### Study participants

The South African Breast Cancer and HIV Outcomes (SABCHO) cohort [21] studied patients recruited at the breast clinic of Chris Hani Baragwanath Academic Hospital (CHBAH), Soweto, South Africa. Participants were consenting women with biopsy-confirmed breast cancer who self-identified as Black African. Exclusion criteria were age < 18 years or current pregnancy. Clinical staging was according to the American Joint Committee on Cancer (AJCC) system [22]. The study was approved by the Human Research Ethics Committee (Medical) at the University of the Witwatersrand (M161116).

### IHC classification of tumors

Histopathological characteristics for 384 patients, obtained from the National Health Laboratory Service (NHLS), included histological type, tumor grade, ER, PR, HER2 scoring and Ki67. All tissues for this study were processed at CHBAH NHLS Laboratory, following College of American Pathologist guidelines. Immunostaining was performed on the Benchmark XT automatic platform. The tumors were classified according to the St Gallen’s Guidelines [5, 13, 23]. The Allred score was used to determine ER/PR status, with a value of 0–2 considered negative, and 3–8 considered positive [8, 24, 25]. Tumors were HER2 positive if they scored 3 + by IHC, or 2 + by IHC with fluorescent in situ hybridization (FISH) confirmation. The Ki67 antibody used was 30–9 (Roche diagnostic, Ventana, USA), and multiple scorers at the same laboratory assessed the Ki67 stains. Percentage of proliferation was determined by visual estimation [17]. The cut-off for the proliferation marker Ki67 is unresolved. The multidisciplinary team at CHBAH uses a Ki67 score of 20% in conjunction with the Allred score, grade and age of patient as a cut off for chemotherapeutic treatment in HR positive breast cancers. We additionally explored cutoffs of 10%, 15%, 20%, 25% and 30%, because of the uncertainty surrounding those values for clinical decision making [12].

We assigned IHC used for clinical decision making as follows: Clin-A (HR + /HER2-/Ki67 ≤ 14%); Clin-B (HR + /HER2-/Ki67 > 14%, or HR + /HER2 + /Ki67 any); Clin-HER2 (HR-/HER2 + /Ki67 any); and Clin-TNC (HR-/HER2-/Ki67 any). The IHC subtyping surrogates were assigned as: A-like (HR + /HER2-/Ki67 ≤ 10%); A- or B-like (HR + /HER2-/10% < Ki67 ≤ 30%); B-like (HR + /HER2-/Ki67 > 30%); B/HER2-like (HR + /HER2 + /Ki67 any); HER2-like (HR-/HER2 + /Ki67 any); TNC (HR-/HER2-/Ki67 any). Both the clinical IHC subtypes as well as the IHC subtyping surrogates were compared with the PAM50 Intrinsic subtypes: luminal-A; luminal-B; HER2-enriched and basal-like (Table 1).

### PAM50 intrinsic subtyping

FFPE blocks were cut into 5 µm serial sections; the area of tumor was identified and marked on an H&E section. If available, primary surgery blocks were preferentially chosen. If the surgery section was unavailable, or if the patient received neoadjuvant chemotherapy or radiation therapy prior to surgery, a biopsy section was used.

RNA was purified from the FFPE sections using the All Prep® DNA/RNA FFPE kit (Qiagen, Hilden, Germany). The RNA concentration was calculated using the optical density at 260 nm on the Nanodrop 2000™ spectrophotometer (Thermo Fisher Scientific, Waltham, MA). The extract was deemed suitable for further analysis if the concentration of RNA was greater than 12.5 ng/µl and the A260/280 ratio was 1.7–2.3. Following RNA extraction, 384 samples were of sufficient quantity and quality for molecular typing.

The PAM50 gene expression was measured on the nCounter SPRINT™ (Nanostring Technologies, Seattle, WA), as per the Prosigna® Breast Cancer Prognostic Gene signature assay Package insert [18]. (The 50 genes and 8 housekeeping genes are shown in supplementary Table S1 and an example of the resultant heat map are shown in supplementary Fig. S1.) nSOLVER 4.0 was used to retrieve the RCF files and perform QC analysis, background subtraction and normalization. Of the 384 samples, 378 passed QC and underwent further analysis; classification of intrinsic subtype was done at Nanostring (Seattle, WA). Quality control (QC) of the data was performed by NanoString Technologies, Inc. using their proprietary software, nSolver. For mRNA samples, as used in this study, QC is performed at a number of stages. Imaging QC flags samples if less than 75% of the imaging surface can be read. Binding density QC calculates the barcodes/micron^2^, samples with binding densities between 0.05 and 1.8 are usable with optimal binding densities being around 1.4 barcodes/micron^2^. The PAM50 panel includes both positive and negative controls which are assessed by geometric mean. Positive controls are synthetic RNA targets, spiked in at known concentrations, that are used to ensure proper hybridization and lack of RNase contamination in the samples and to establish limits of detection (the 0.5 fM positive control must be more than 2 standard deviations above the mean of the negative controls to pass QC). Positive controls are also used in normalization QC by generating scaling factors that must be between 0.3 and 3 to pass QC. Negative controls are probes for which no known target exists in biological samples and are used to establish background levels of detection.

### Statistical analysis

Continuous variables were assessed for normality using the Shapiro-Wilks test. The data were described by mean ± standard deviation for normally distributed variables and median (interquartile range) for non-normally distributed variables. Categorical variables were described as frequencies and percentages. Statistical analyses were done using STATA v14.2 (College Station, Texas). Significance between the groups was determined using Pearson’s χ^2^ test or the Kruskall Wallis rank test, and post hoc analysis using Dunn’s Pairwise Comparison test. A *p*-value < 0.05 was considered significant. Agreement in subtype call between the IHC and PAM50 subtyping methods was assessed using the kappa statistic. To allow for comparable groups with this method, the IHC results were classified as follows: Clin-A (HR + /HER2-/Ki67 ≤ 14%), Clin-B (HR + /HER2-/Ki67 > 14%), Clin-HER2 (HR any/HER2 + /Ki67 any) and TNC (HR-/HER2-/Ki67 any).

## Results

### Characteristics of the study cohort

The clinicopathological characteristics are shown in Table 2. The mean age of study participants was 49.7 years. Most patients had stage II or III cancers, and were more likely to have grade-2 or -3 tumors between 20 and 50 mm (AJCC T2), with some nodal involvement.

**Table 2:** Clinicopathological characteristics of the study cohort

	*n* = 378	%
*Age* mean (± SD)	49.71 yr (± 11.02)	
*Stage at diagnosis*		
Stage I	13	3.44
Stage II	152	40.21
Stage III	173	45.77
Stage IV	40	10.58
*Grade (n* = *374)*		
Grade 1	24	6.42
Grade 2	207	55.35
Grade 3	143	38.24
Tumor stage* (n = *378)*		
T1	35	9.26
T2	168	44.44
T3	77	20.37
T4	98	25.93
*Histology Diagnosis or Histological subtype (n* = *372)*		
Invasive Ductal	329	88.4
Invasive Lobular	15	4.0
Invasive Mucinous	10	2.7
Other**	18	4.8
*Nodal Involvement (n* = *375)*		
Absent (0 nodes)	82	21.87
1–3 nodes	175	46.67
4–9 nodes	88	23.47
10 + nodes	30	8.00
*ER (n* = *378)*		
Positive	293	77.51
Negative	85	22.49
*PR (n* = *378)*		
Positive	267	70.63
Negative	111	29.37
*HER2 (n* = *378)*		
Positive	122	32.28
Negative	256	67.72
*Allred score (n* = *369)*		
Negative (Allred = 0,1,2)	66	17.87
Low (Allred = 3,4)	30	8.13
Intermediate (Allred = 5,6)	25	6.78
High (Allred = 7,8)	248	67.21
*Ki67 (n* = *375)*		
0% < Ki67 ≤ 10%	33	8.80
10% < Ki67 ≤ 15%	12	3.20
15% < Ki67 ≤ 20%	30	8.00
20% < K67 ≤ 25%	10	2.67
25% < Ki67 ≤ 30%	40	10.67
30–100%	250	66.67
*PAM50 (n* = *378)*		
Luminal-A	73	19.31
Luminal-B	123	32.54
HER2-enriched	89	23.54
Basal-like	93	24.60
*IHC Clinical (n* = *377)*		
Clin-A (HR + /HER2-/Ki67 ≤ 14%)	26	6.90
Clin-B (HR + /HER2-/Ki67 > 14% or HR + /HER2 +)	274	72.68
Clin-HER2 (HR-/HER2 +)	20	5.31
Clin-TNC (HR-/HER2-)	57	15.11
IHC Clinical with grading*** (n = *374)*		
Low grade Clin-A	23	6.15
High grade Clin-A	2	0.53
Low grade Clin-B	178	47.59
High grade Clin-B	94	25.13
Clin-HER2	20	5.35
Clin-TNC	57	15.24
*IHC Analysis (n* = *377)*		
A-like (HR + /HER2-/Ki67 ≤ 10%)	26	6.90
A-or B- like (HR + /HER2-/10% < Ki67 ≤ 30%)	55	14.59
B-like (HR + /HER2-/ Ki67 > 30%)	117	31.03
B/HER2-like (HR + /HER2 + /Ki67 any)	102	27.06
HER2-like (HR-/HER2 + /Ki67 any)	20	5.31
TNC (HR-/HER2-/Ki67 any)	57	15.11

*Tumor staging was based on clinical staging

**“Other” histological subtypes were 1 apocrine, 3 medullary, 1 metaplastic, 1 neuroendocrine, 5 papillary, 3 squamous, and 4 tubular invasive carcinomas

***IHC clinical with grading—the clinical immunotypes were subanalyzed with low (1 or 2) or high (3) grade

*ER* estrogen receptor, *PR* progesterone receptor, *IHC* immunohistochemistry, *HR* hormone receptor, *HER2* human epidermal growth factor receptor 2, *TNC* triple negative (breast) cancer

**Fig. 1:**
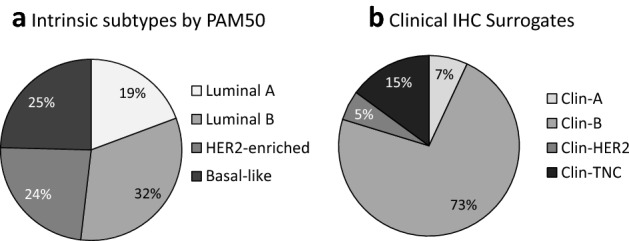
Cohort subtypes by **a** Intrinsic subtyping by the PAM50 assay and **b** Clinical IHC subtypes. **a** The percentage distribution of intrinsic subtypes was luminal-B (32.5%, *n* = 123), basal-like (24.6%, *n* = 93), HER2-enriched (23.6%, *n* = 89), and luminal-A (19.3%, *n* = 73). However, using immunohistochemistry **b** as a surrogate for intrinsic subtypes, only 6.9% (*n* = 26) of the samples tested were categorized as Clin-A (ER/PR pos, HER2 neg and Ki67 ≥ 14%). Most of the samples (72.7%, *n* = 274) were Clin-B, i.e. ER/PR pos, HER2 neg, Ki67 > 14% or ER/PR pos, HER2 pos, any Ki67. A small minority (5.3%, *n* = 20) were Clin-HER2 (ER/PR neg, HER2 pos). The TNC (ER/PR neg, HER2 neg, Ki67 any) accounted for the remaining 15.1% (*n* = 57)

The intrinsic subtyping distribution by the PAM50 assay was 19.3% luminal-A (*n* = 73), 32.5% luminal-B (*n* = 123), 23.5% HER2-enriched (*n* = 89) and 25.6% basal-like (*n* = 93) (Fig. 1a, Table 2). When classified by IHC, most patients (79.6%) were HR positive (with, or without, HER2) (Fig. 1b). Although the intrinsic subtypes (Fig. 1a) show roughly equal numbers of luminal-A (19.3%), luminal-B (32.5%), HER2-enriched (23.5%) and basal-like (24.6%) subtypes, the clinical IHC results show a massive predominance of Clin-B subtype (72.7%), and only 6.9% Clin-A, 5.3% Clin-HER2 and 15.1% Clin-TNC. High grade (3) Clin-A subtype, treated as Clin-B by the multidisciplinary team, only accounted for 0.53% (Table 2) of the total cohort, and did not meaningfully affect the concordance with the molecular subtypes.

### Comparison of immunohistochemistry and intrinsic subtypes

**Fig. 2:**
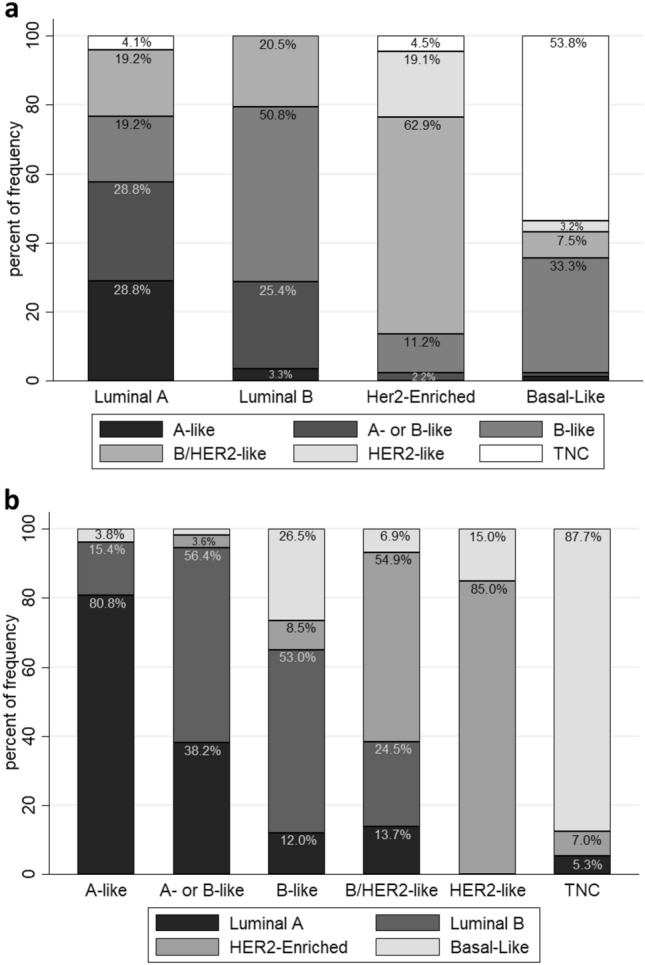
Comparison between immunohistochemical subtypes and molecular subtypes. **a** Frequency of immunohistochemical analysis subtypes (y-axis) within the PAM50 molecular defined tumor subtypes (x-axis). The luminal-A intrinsic subtype had the greatest spread of IHC types, with 28.8% IHC A-like, 28.8% IHC A- or B-like, 19.2% IHC B-like, 19.2% IHC B/HER2, 0% HER2-like (HR-/HER2 +) and 4.1% TNC. The luminal B intrinsic subtype was mainly B-like (50.8%), with 25.4% A- or B- like, and 20.5% B/HER2-like. The HER2-enriched intrinsic subtype was mainly B/HER2-like (62.9%), followed by HER2-like (HR-/HER2 + , 19.1%), and the basal-like was mainly TNC (53.8%) or B-like (33.3%). **b** Intrinsic subtype frequency (y-axis) concordance with each IHC-Analysis subgroup classification (x-axis). Most of the A-like (HR + /HER2-/Ki67 ≤ 10%) group had the luminal-A subtype (80.8%). The A- or B- like group (HR + /HER2-/10% < Ki67 ≤ 30%) had slightly more luminal-B (56.4%) then luminal-A (38.2%) subtypes. The B-like group (HR + /HER2-/Ki67 < 30%) had mainly luminal-B subtypes (53%). B/HER2-like (HR + /HER2 +) tumors were mainly HER2-enriched (54.9%), as was expected, while the HER2 group (HR-/HER2 +) was predominantly HER2-enriched (85%). The TNC group had overwhelming basal-like subtypes (87.7%)

The luminal-B intrinsic subtype and the IHC B-like (Fig. 2a) were highly concordant. The intrinsic HER2-enriched showed the best concordance with the IHC B/HER2-like and the HR-/HER2-like (62.9% and 19.1%, respectively), while the intrinsic basal-like was most concordant with the IHC TNC (53.8%). Immunohistochemistry currently classifies the B/HER2-like as B-like tumors because they are HR positive but it may be more appropriate to classify these B/HER2-like tumors as HER2 positive tumors and to treat them accordingly. The intrinsic luminal-A subtype was not strongly associated with any one IHC subtype, raising questions about appropriate Ki67 cutoff values.

By comparison, the IHC-like groups were well reflected by the intrinsic subtypes (Fig. 2b). The A-like group was mainly composed of luminal-A intrinsic subtypes; A- or B- like was primarily distributed between luminal-A (38.2%) and luminal-B (56.4%) intrinsic subtypes, and the IHC B-like was mainly comprised of luminal-B. The HR positive/HER2 + (B/HER2-like) group consisted mainly of the intrinsic HER2-enriched subtype, followed by the luminal-B subtype. The HR negative / HER2 positive (HER2-like) group was predominantly HER2-enriched, and the TNC group mainly basal-like, as expected.

### Characteristics by intrinsic subtype

Expression of the proliferation marker, Ki67, was lowest in luminal-A tumors [20% (10–32.5%)], highest in the basal-like subtype [70% (50–80%)], and intermediate in the luminal-B [40% (30–55%)] and HER2-enriched [50% (40–62.5%)] subtypes, as expected (Table 3). Categorical analysis of Ki67 expression showed that the luminal-A tumors had the greatest spread, while close to 80% of the luminal-B tumors had Ki67 levels > 30%. The HER2-enriched and basal-like tumors expressed Ki67 at high values (over 30%), as expected. The Allred scores in luminal-A and luminal-B subtypes were predominantly high (scores of 7,8), while HER2-enriched subtypes had a greater spread of HR expression scores and basal-like subtypes were mainly negative or low scoring (Table 3).

**Table 3:** Tumor features by intrinsic subtype

	Luminal-A	Luminal-B	HER2-Enriched	Basal-Like	*p*- value^*^
*Ki67 (n* = *375)*					
% Ki67 median (IQR)	20 (10–32.5)	40 (30–55)	50 (40–62.5)	70 (50–80)	< 0.001
Ki67 Categories					
< 10; *n* = 18	14 (19.44%)	1 (0.82%)	3 (3.41%)	0 (0%)	
10–15; *n* = 15	10 (13.89%)	3 (2.46%)	1 (1.14%)	1 (1.08%)	
15–20; *n* = 14	8 (11.11%)	5 (4.1%)	1 (1.14%)	0 (0%)	
20–30; *n* = 38	15 (20.83%)	16 (13.11%)	6 (6.82%)	1 (1.08%)	
> 30; *n* = 290	25 (34.72%)	97 (79.51%)	77 (87.5%)	91 (97.85%)	
*Allred (n* = *369)*					< 0.001
Negative (0,1,2)	2 (2.78%)	0	16 (18.60%)	48 (54.55%)	
Low (3,4)	2 (2.78%)	0	9 (10.47%)	19 (21.59%)	
Intermediate (5,6)	3 (4.17%)	3 (2.44%)	14 (6.88%)	5 (5.68%)	
High (7,8)	65 (90.28%)	120 (97.6%)	47 (54.65%)	16 (18.18%)	
*T Staging (n* = *378)*					< 0.001
T1, *n* = 35	13 (17.81%)	8 (6.50%)	7 (7.87%)	7 (7.53%)	
T2, *n* = 168	38 (52.05%)	68 (55.28%)	25 (28.09%)	37 (39.78%)	
T3, *n* = 77	8 (10.96%)	19 (15.45%)	24 (26.97%)	26 (27.96%)	
T4, *n* = 98	14 (19.18%)	28 (22.76%)	33 (37.08%)	23 (24.73%)	
*Age at diagnosis*	48 (42–59)	49 (42–60)	46 (41–52)	48 (42–56)	0.297
*Age below and above 50 years*					0.679
< 50 years	39 (18.48%)	65 (30.81%)	54 (25.59%)	53 (25.12%)	
≥ 50 years	34 (20.36%)	58 (34.73%)	35 (20.96%)	40 (23.95%)	
*Stage at diagnosis*					0.004
Early Stage	43 (58.9%)	55 (44.7%)	27 (30.3%)	40 (43.0%)	
Late Stage	30 (41.1%)	68 (55.3%)	62 (69.7%)	53 (57.0%)	
*Grade (n* = *374)*					< 0.001
Grade 1, *n* = 24	17 (23.6%)	4 (3.3%)	3 (3.4%)	0	
Grade 2, *n* = 207	54 (75.0%)	81 (66.4%)	45 (55.7%)	23 (25.0%)	
Grade 3, *n* = 143	1 (1.4%)	37 (30.3%)	36 (40.9%)	69 (75.0%)	
*Nodal involvement*	56 (76.7%)	98 (79.7%)	74 (83.2%)	68 (73.1%)	0.400
*Histological Diagnosis (n* = *372)*					< 0.001
Invasive Ductal	55 (76.4%)	111 (91.7%)	79 (90.8%)	84 (91.3%)	
Invasive Lobular	8 (11.1%)	5 (4.1%)	2 (2.3%)	0	
Invasive Mucinous	5 (6.9%)	4 (3.3%)	1 (1.2%)	0	
Other	4 (5.6%)	1 (0.8%)	5 (5.8%)	8 (8.7%)	

*Median (IQR) values compared using the Kruskal–Wallis equality-of-populations rank test; frequencies compared using Pearson’s χ2 test or Fisher's exact test if any groups had frequencies of 5 or less

Luminal-A (69.9%) and luminal-B (61.8%) subtypes were more likely to have lower T stages (T1 or T2), compared to HER2-enriched (36.0%) and basal-like (47.3%) subtypes. All intrinsic subtypes had T4 tumors, indicative of the late stage at presentation in this setting (Table 3). Tumors with a luminal subtype were more likely to be of lower grade (grade 1 or 2) than basal-like subtypes (75.0% grade 3). Histologically, only the luminal-A subtypes had a significant proportion of invasive lobular carcinomas (11.1%) and invasive mucinous carcinomas (6.9%). Age and nodal involvement were not associated with intrinsic subtype in this cohort (Table 3).

### Comparisons of Ki67 cutoff levels

**Fig. 3:**
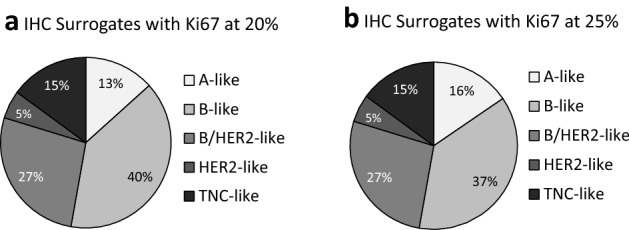
IHC Analysis subtypes with different Ki67 cutoff values to distinguish between IHC A-like and IHC B-like groups. IHC A-like with Ki67 ≥ 20% and IHC B-like with Ki67 < 20% is shown in (**a**); while IHC A-like with Ki67 ≥ 25% and IHC B-like with Ki67 < 25% is shown in (**b**). When the cutoff for Ki67 between IHC A-like and B-like is at 20% (**a**), 13.3% (*n* = 50) of the ER/PR pos, HER2 neg cohort is A-like and 39.5% (*n* = 148) is B-like. When the cutoff is at 25% (**b**), 15.5% (*n* = 58) of the ER/PR pos, HER2 neg cohort is A-like and 37.3% (*n* = 140) is B-like. When the ER/PR pos, HER2 pos (B/HER2-like) was classified separately from B-like group, it comprised 26.9% (*n* = 101) of the cohort. The HER2 (ER/PR neg, HER2 pos) group made up 5.3% (*n* = 20) of the cohort, and 14.9% (*n* = 56) of the cohort was triple negative (ER/PR neg, HER2 neg)

The kappa test was used to compare the classification of the luminal subtype using IHC and PAM50 based on Ki67 levels (Supplementary Table S2). The IHC groups were split into luminal-A and luminal-B subtypes using Ki67 cutoffs of 10%, 15%, 20%, 25% and 30% and the kappa statistic was used to compare these classifications to the subtypes assigned by the PAM50 analysis. The agreement between the methods ranged from 43 to 49%. The best concordance of the IHC and intrinsic subtypes, was when the cutoff was at 25% Ki67 (κ = 0.128, *p* = 0.003) and the worst at a cutoff of 10% (κ = 0.079, *p* = 0.033) (Supplementary Table S2). Thus, a Ki67 cutoff of 25% appears best for separating the luminal-A and -B subtypes in our setting. Using the 25% cutoff results in 15.5% IHC A-like and 37.3% B-like (Fig. 3b), closer in value to the intrinsic subtype proportions of luminal-A (19%) and luminal-B (32%) (Fig. 1a) than the current clinical cutoff of 14% (Fig. 1b). Moreover, when IHC HR + /HER2 + samples are separated from the Clin-B (Fig. 1b) into the B/HER2-like group (Fig. 3), the B-like group becomes smaller, but the B/HER2-like group (26.9%) and HER2-like group (5.3%) together, are more reflective of the HER2-enriched intrinsic subtype (Fig. 1b).

## Discussion

The ability to diagnose breast cancer subtypes accurately and appropriately, fundamentally affects cancer treatment decisions. PAM50 is widely used for molecular diagnosis of breast cancer subtypes in high income countries (HICs) [26] because its results are reproducible and unaffected by inter- and intra-laboratory variability [27]. Within resource-constrained settings, IHC is used as a proxy for intrinsic subtypes because it is less expensive, the infrastructure to run IHC assays is widespread, and it requires less “hands-on” technical expertise than the PAM50 assay. We thus need accurate and population-specific information to assign proxies that optimize concordance calibration with the PAM50 intrinsic subtyping findings.

We found that the luminal-A intrinsic subtype had the greatest spread of IHC-analysis subgroups; the A-like IHC group was mainly composed of luminal-A subtype. This observation suggests that the currently used 14–20% Ki67 cutoff in South Africa may be too low. If the Ki67 cutoff were increased to 20–25%, the IHC A-like and B-like distribution would more accurately reflect the intrinsic subtypes. Subtyping strongly affects treatment options. Patients with luminal-A subtypes are likely to benefit from primary endocrine therapies in place of chemotherapy as first choice systemic treatment, whereas the benefits of chemotherapy to patients with luminal-B subtypes may offset chemotherapy side effects [28]. The ambiguity in the Ki67 cutoff is not unique to the South African public health care system. German guidelines state that primary invasive tumors that are HR + , HER2- are considered low risk if Ki67 ≤ 10%, high risk if ≥ 25%, and intermediate risk if 10–25% as Ki67 does not differentiate risk groups accurately in this range [12]. By contrast, the 14% cutoff was the best to distinguish between luminal-A and luminal-B in Spanish and Italian patients using Prosigna™ assays [29]. These results reinforced the original PCR findings of Cheang et al. [10] that the 14% cutoff was optimal. However, like Noske et al. [12], we observed better concordance at higher Ki67 levels.

In HICs, where most breast cancers are diagnosed in early stages, the ASCO recommendations [14] suggested that PAM50 could be used to inform chemotherapy decisions, much better than IHC in node negative luminal subtypes. Pu et al. [30] found that survival rates were consistently worse in the luminal-B subtype, irrespective of menopausal status. The 2019 St Gallen report recommended that patients with ER ≥ 1% receive endocrine therapy, although it might have limited benefits [28]. This recommendation is in line with the South African policy, which regards ER or PR ≥ 1% as hormone receptor positive [31]. The Allred score shows ER and/or PR expression is high in luminal-A and luminal-B subtypes, as expected. The PAM50 basal-like subtype was predominantly negative for the Allred score, but also had a portion of low (3,4) Allred scores. This second finding is interesting, as it may suggest that the Allred cutoff to distinguish between A-like, B-like IHC subtypes and TNC subtypes could be increased to an Allred score ≤ 4. A larger study is needed to confirm this.

Most tumors of the HER2-enriched luminal subtype are assigned to the B/HER-like IHC-analysis group. This finding is obvious when looking at the Allred score, where most of the HER2-enriched luminal subtypes had high HR positivity. While the multidisciplinary teams follow the St Gallen’s recommendations and treat HR + /HER2 + as Clin-B, the PAM50 intrinsic subtypes do not make this subtle distinction. In South African public health care, patients in this group received adjuvant endocrine therapy until 2019, when anti-HER2 therapies were introduced. A mere 19% of the HER2 enriched subtype would be HR negative and would not benefit from endocrine therapy.

Patient subtyping should be interpreted cautiously. Mistaking luminal-A patients for luminal-B may result in overtreatment with chemotherapy. Confusion of HER2 with luminal-B subtypes may result in under treatment by HER2 targeted therapy (e.g., trastuzumab) and/or overtreatment endocrine therapy [32]. Trastuzumab is expensive and inconsistently available in the South African public sector [33], so the option of using endocrine therapies if trastuzumab is unavailable would be an advantage for HER2 positive patients.

A Swedish cohort, [34] found 81–85% concordance between molecular luminal-A and IHC-A subtypes. However, 35–52% of their luminal-B intrinsic subtypes were classified as IHC-A. Ki67 distinguished between good and bad prognostic groups with node negative cancer, but its use is very controversial [34]. Lundgren et al. [35] found that concordance with luminal subtypes improved when histological grade was included. Well differentiated tumors (grade 1) tended to have low Ki67 levels [12]. Intermediate (grade 2) and poorly differentiated tumors (grade 3) had higher Ki67 levels and a wider range of Ki67 values [12]. In our study histological grades were generally high, so including grade with clinical IHC subtype had a negligible effect on concordance.

Previously, women of African ancestry were thought to have fewer hormone receptor positive breast cancers than women of European ancestry. West African women and African-American women appear more likely to have TNC cancers [36–41]. However, research has shown that most sub-Saharan Africans (South African, Kenyan, Sudanese) [15, 21, 42–45] have HR positive cancers. In our cohort, 79.5% were HR positive, and more likely to be B-like (i.e., HR positive, high Ki67), even when the cutoff of Ki67 is 30%. Such cancers are more aggressive and have a poorer prognosis than those classified as luminal-A or IHC A-like.

Because our study was part of a HIV outcome study, HIV positive and HIV negative cases were age matched within a 5 year band. Our study participants were therefore younger (49.9 years ± 11 years) than South African women with breast cancer on average. Younger patients are thought to have more clinically aggressive disease and poorer outcomes. Korean breast cancer patients are much more likely to be premenopausal than others [46], and this younger population shows poorer outcomes. Sub-Saharan Africa shows huge disparities in IHC subtyping [47]. In Uganda, breast cancer patients had mean age of 45, with IHC of 38% A or B; 5% B/HER2; 22% HER2 and 34% TNC [48]. Two separate Nigerian groups found very different IHC expression: a study in Ibadan, found 77.6% A or B; 2.6% B/HER2; 4% HER2 and 15.8% TNC [49]; while a different study in Lagos found 38% HR pos; 18.3% HER2 pos and 47.4% TNC [50]. Patients in Mozambique [51], had IHC of 51% A or B; 24% HER2 pos and 25% TNC; and Angola reported 25.7% A-like; 19.3% B-like; 7.9% B/HER2; 15.7% HER2-like and 31.4% TNC [52]; while in Zimbabwe, the IHC was 68% HR positive and 17% TNC [53]. Work on 985 participants in South Africa showed 13.8% A-like; 43.9% B-like, 19.0% B/HER2; 6.0% HER2-like and 15.3% TNC, although this work included individuals of different ethnicities [19]. Recent work in South Africa [20] found that black South Africans had expression of about 49–53% HR + /HER2- (A- or B-like), 13–18% HR + /HER2 + (B/HER2-like), 7–12% HR-/HER2 + and 23–27% TNC, regardless of age. By comparison, South African whites had 30–65% HR + /HER2- (A- or B-like), 9–29% HR + /HER2 + , 4–13% HR-/HER2 + and 14–29% TNC. White women under 40 had higher expression of the more aggressive TNC and HER2 tumors, while women over 60 had more A-like and B-like tumors. Our results, with exclusively black participants, did not show differences in between the distribution of subtypes with age, which is consistent with the results found by Achilonu et al. [20].

Limitations of this study include the small sample size and lower age of participants. This may have artificially increased the proportion of HER2 positive tumors. However, these limitations may have had reduced impact on the main focus of this study; which was the discordance between PAM50 intrinsic subtyping and IHC surrogates.

Our study is, as far as we know, the first to compare IHC with PAM50 in black southern African women. Most of our study participants had hormone receptor positive breast cancer, and even tumors with the HER2-enriched subtype were more likely to be HR positive than HR negative. PAM50 is widely used for breast cancer subtyping, with IHC often used in resource constrained settings. The cost and labor of the PAM50 method make it prohibitive for the South African public health care sector and its inability to distinguish between HER2-positive B-subtypes and HR negative/HER2 positive subtypes must also give pause. We found the lowest concordance between molecular and IHC subtyping for the luminal-A group and recommend raising the cutoff for Ki67 to 20–25% to distinguish between A-like and B-like tumors, to better reflect the luminal subtypes.

## Supplementary Information

Below is the link to the electronic supplementary material.

Supplementary file1 (DOCX 112 KB)

Supplementary file2 (DOCX 33 KB)

Supplementary file3 (DOCX 23 KB)

## Data Availability

Data are available upon reasonable request.

## Abbreviations

AJCC: American Joint Committee on Cancer
CHBAH: Chris Hani Baragwanath Academic Hospital
ER: Estrogen receptor
FFPE: Formalin-fixed paraffin embedded
FISH: Fluorescent in situ hybridization
HER2: Human epidermal growth factor receptor 2
HIC: High-income countries
HR: Hormone receptor
IHC: Immunohistochemistry
Neg: Negative
Pos: Positive
PR: Progesterone receptor
SABCHO: South African Breast Cancer and HIV Outcomes
TNC: Triple negative breast cancer
