# Supplementary material for: Discordance between PAM50 intrinsic subtyping and immunohistochemistry in South African women with breast cancer
Source: Breast Cancer Res Treat. Author manuscript; Available in PMC 2023 May 1. (PMC10147771; doi:10.1007/s10549-023-06886-3)
Supplement: Supp file 2 [file NIHMS1880146-supplement-Supp_file_2.docx]

**Discordance between PAM50 intrinsic subtyping and Immunohistochemistry in South African Women with Breast Cancer**

Authors:

Thérèse Dix-Peek, Boitumelo P. Phakathi, Eunice J. van den Berg, Caroline Dickens, Tanya N. Augustine, Herbert Cubasch, Alfred I. Neugut, Judith S. Jacobson, Maureen Joffe, Paul Ruff, Raquel A.B. Duarte

Table S1. PAM50 gene list **(**Wallden et al., 2015;

| **Type** | **Gene Card** | **Accession number** | **Gene Name** |
| --- | --- | --- | --- |
| Endogenous | BAG1 | NM_004323.3 | BCL2-associated athanogene |
| Endogenous | BCL2 | NM_000633.2 | B-cell CLL/lymphoma 2, nuclear gene encoding mitochondrial protein |
| Endogenous | BLVRA | NM_000712.3 | Biliverdin reductase A |
| Endogenous | CXXC5 | NM_016463.5 | CXXC finger 5 |
| Endogenous | ESR1 | NM_000125.2 | Estrogen receptor 1 |
| Endogenous | FOXA1 | NM_004496.2 | Forkhead box A1 |
| Endogenous | GPR160 | NM_014373.1 | G protein-coupled receptor 160 |
| Endogenous | MAPT | NM_016835.3 | Microtubule-associated protein tau |
| Endogenous | MDM2 | NM_006878.2 | Mdm2, transformed 3T3 cell double minute 2, p53 binding protein (mouse) |
| Endogenous | MLPH | NM_024101.4 | Melanophilin |
| Endogenous | MMP11 | NM_005940.3 | Matrix metallopeptidase 11 (stromelysin 3) |
| Endogenous | MYC | NM_002467.3 | v-myc myelocytomatosis viral oncogene homolog (avian) |
| Endogenous | NAT1 | NM_000662.4 | N-acetyltransferase 1 (arylamine N-acetyltransferase) |
| Endogenous | PGR | NM_000926.2 | Progesterone receptor |
| Endogenous | SLC39A6 | NM_012319.2 | Solute carrier family 39 (zinc transporter), member 6 |
| Endogenous | ERBB2 | NM_004448.2 | V-erb-b2 erythroblastic leukemia viral oncogene homolog 2, neuro/glioblastoma derived oncogene homolog (avian) |
| Endogenous | FGFR4 | NM_002011.3 | Fibroblast growth factor receptor 4 |
| Endogenous | GRB7 | NM_005310.2 | Growth factor receptor-bound protein 7 |
| Endogenous | TMEM45B | NM_138788.3 | Transmembrane protein 45B |
| Endogenous | ACTR3B | NM_001040135.1 | ARP3 actin-related protein 3 homolog B (yeast) |
| Endogenous | CDH3 | NM_001793.3 | Cadherin 3, type 1, P-cadherin (placental) |
| Endogenous | EGFR | NM_005228.3 | Epidermal growth factor receptor (erythroblastic leukemia viral (v-erb-b) oncogene homolog, avian) |
| Endogenous | FOXC1 | NM_001453.1 | Forkhead box C1 |
| Endogenous | KRT14 | NM_000526.3 | Keratin 14 (epidermolysis bullosa simplex, Dowling-Meara, Koebner) |
| Endogenous | KRT17 | NM_000422.1 | Keratin 17 |
| Endogenous | KRT5 | NM_000424.2 | Keratin 5 (epidermolysis bullosa simplex, Dowling-Meara/Kobner/Weber-Cockayne types) |
| Endogenous | MIA | NM_006533.1 | Melanoma inhibitory activity |
| Endogenous | PHGDH | NM_006623.2 | Phosphoglycerate dehydrogenase |
| Endogenous | SFRP1 | NM_003012.3 | Secreted frizzled-related protein 1 |
| Endogenous | ANLN | NM_018685.2 | Anillin, actin binding protein (scraps homolog, Drosophila) |
| Endogenous | BIRC5 | NM_001168.2 | Baculoviral IAP repeat-containing 5 (survivin) |
| Endogenous | CCNB1 | NM_031966.2 | Cyclin B1 |
| Endogenous | CCNE1 | NM_001238.1 | Cyclin E1 |
| Endogenous | CDC20 | NM_001255.1 | CDC20 cell division cycle 20 homolog (S. cerevisiae) |
| Endogenous | CDC6 | NM_001254.3 | CDC6 cell division cycle 6 homolog (S. cerevisiae) |
| Endogenous | CDCA1 | NM_145697.1 | Cell division cycle associated 1 |
| Endogenous | CENPF | NM_016343.3 | Centromere protein F, 350/400ka (mitosin) |
| Endogenous | CEP55 | NM_018131.3 | Centrosomal protein 55kDa |
| Endogenous | EXO1 | NM_006027.3 | Exonuclease 1 |
| Endogenous | KIF2C | NM_006845.2 | Kinesin family member 2C |
| Endogenous | KNTC2 | NM_006101.1 | Kinetochore associated 2 |
| Endogenous | MELK | NM_014791.2 | Maternal embryonic leucine zipper kinase |
| Endogenous | MKI67 | NM_002417.2 | Antigen identified by monoclonal antibody Ki-67 |
| Endogenous | MYBL2 | NM_002466.2 | v-myb myeloblastosis viral oncogene homolog (avian)-like 2 |
| Endogenous | ORC6L | NM_014321.2 | Origin recognition complex, subunit 6 like (yeast) |
| Endogenous | PTTG1 | NM_004219.2 | Pituitary tumor-transforming 1 |
| Endogenous | RRM2 | NM_001034.1 | Ribonucleotide reductase M2 polypeptide |
| Endogenous | TYMS | NM_001071.1 | Thymidylate synthetase |
| Endogenous | UBE2C | NM_007019.2 | Ubiquitin-conjugating enzyme E2C |
| Endogenous | UBE2T | NM_014176.1 | Ubiquitin-conjugating enzyme E2T (putative) |
| Reference | ACTB | NM_001101.2 | Actin, beta |
| Reference | GUSB | NM_000181.1 | Glucuronidase, beta |
| Reference | MRPL19 | NM_014763.3 | Mitochondrial ribosomal protein L19 |
| Reference | PSMC4 | NM_006503.2 | Proteasome (prosome, macropain) 26S subunit, ATPase, 4 |
| Reference | PUM1 | NM_001020658.1 | Pumilio RNA-binding family member 1 |
| Reference | RPLP0 | NM_001002.3 | Ribosomal protein, large, P0 |
| Reference | SF3A1 | NM_005877.4 | Splicing factor 3a, subunit 1, 120kDa |
| Reference | TFRC | NM_003234.1 | Transferrin receptor (p90, CD71) |
