# Supplementary material for: Discordance between PAM50 intrinsic subtyping and immunohistochemistry in South African women with breast cancer
Source: Breast Cancer Res Treat. Author manuscript; Available in PMC 2023 May 1. (PMC10147771; doi:10.1007/s10549-023-06886-3)
Supplement: Supp file 3 [file NIHMS1880146-supplement-Supp_file_3.docx]

**Discordance between PAM50 intrinsic subtyping and Immunohistochemistry in South African Women with Breast Cancer**

Authors:

Thérèse Dix-Peek, Boitumelo P. Phakathi, Eunice J. van den Berg, Caroline Dickens, Tanya N. Augustine, Herbert Cubasch, Alfred I. Neugut, Judith S. Jacobson, Maureen Joffe, Paul Ruff, Raquel A.B. Duarte

### Supplementary Table 1: Comparisons for PAM50 and IHC luminal subtypes based on Ki67 cutoff levels

| Ki67 cutoff | Agreement | Expected agreement | Kappa statistic | p-value |
| --- | --- | --- | --- | --- |
| 10% | 47.50% | 43.01% | 0.079 | 0.033 |
| 15% | 49.17% | 42.33% | 0.119 | 0.004 |
| 20% | 47.92% | 40.97% | 0.118 | 0.006 |
| 25% | 47.92% | 40.28% | **0.128** | **0.003** |
| 30% | 43.33% | 38.33% | 0.081 | 0.044 |
