# Supplementary material for: Discordance between PAM50 intrinsic subtyping and immunohistochemistry in South African women with breast cancer
Source: Breast Cancer Res Treat. 2023 Mar 3;199(1):1–12. doi: 10.1007/s10549-023-06886-3 (PMC10147771; doi:10.1007/s10549-023-06886-3)
Supplement: Supplementary file 1 [file 10549_2023_6886_MOESM1_ESM.docx]

**Discordance between PAM50 intrinsic subtyping and Immunohistochemistry in South African Women with Breast Cancer**

Authors:

Thérèse Dix-Peek, Boitumelo P. Phakathi, Eunice J. van den Berg, Caroline Dickens, Tanya N. Augustine, Herbert Cubasch, Alfred I. Neugut, Judith S. Jacobson, Maureen Joffe, Paul Ruff, Raquel A.B. Duarte

**Figure S1: Representative heatmap of intrinsic subtypes**

Twenty randomly chosen luminal-A, luminal-B, HER2-enriched and basal-like tumors are represented.  The luminal-A, luminal-B, basal-like cluster with each other. The HER2-enriched subtypes have 2 clusters, one where they cluster with the luminal-subtypes (indicative of HR-positive/HER2 positive subtypes), and a second cluster indicative of HR-negative, HER2-positive subtypes. Upregulation of genes is represented by green and downregulation represented by red.
